# Pseudo Alopecia Areata Caused by Skull-caps with Metal Pin Fasteners used by Orthodox Jews in Israel

**DOI:** 10.1080/10446670310001642131

**Published:** 2003

**Authors:** Chaim Yosefy, Meir Ronnen, Dennis Edelstein

**Affiliations:** 1 Clalit Healthcare, Ganey Barnea, Ashkelon Israel and the Barzilai Medical Center Campus Ben-Gurion University of the Negev Ashkelon Israel; 2 Department of Dermatology Barzilai Medical Center Campus Ben-Gurion University of the Negev Ashkelon Israel; 3 Department of Dermatology Remez Medical Center Rehovot Israel

## Abstract

*Background*: Alopecia Areata (AA) is a disease characterized by hair loss that is widely believed to be autoimmune in origin. Thus treatment is generally aimed in this direction using immune inhibitors such as steroids and PUVA.

*Objective*: To describe a variant of AA, Pseudo Alopecia Areata, caused by a particular cupola pin holder (tic-tac) and to offer a non-pharmacological treatment option (NPT).

*Methods*: A prospective open label study in 37 Jewish religious patients (34 males, 3 females, mean 35 ± 2 years), previously diagnosed and treated for scalp AA were randomly referred to one of the three NPT intervention methods: small cupola held by two pins, large cupola held by one pin and similar cupola held by a different pin.

*Results*: Three of the ten patients (33.3%) from the first group developed secondary AA from the additional pin. No changes were seen in the second group. Ten of the seventeen patients (58.8%) from the third group achieved immediate improvement subsequent to replacing the original pin with a new one on a larger cupola.

*Conclusions*: Conservative pharmacological treatment failed to repair the lesions. The addition of a second pin caused an additional lesion. In contrast, replacing the cupola with a larger one and the original pin-fastener with a different type, successfully reduced the lesions.


*Clinical & Developmental Immunology*, June–December 2003 VOL. 10 (2–4), pp. 193–195

Taylor & Francis healthsciences

### Pseudo Alopecia Areata Caused by Skull-caps with Metal Pin Fasteners used by Orthodox Jews in Israel*

CHAIM YOSEFY[a,†], MEIR RONNEN[b] and DENNIS EDELSTEIN[c]

[a]*Clalit Healthcare, Ganey Barnea, Ashkelon, Israel and the Barzilai Medical Center Campus, Ben-Gurion University of the Negev, Ashkelon, Israel;* [b]*Department of Dermatology, Barzilai Medical Center Campus, Ben-Gurion University of the Negev, Ashkelon, Israel;* [c]*Department of Dermatology, Remez Medical Center, Rehovot, Israel*

*Background*: Alopecia Areata (AA) is a disease characterized by hair loss that is widely believed to be autoimmune in origin. Thus treatment is generally aimed in this direction using immune inhibitors such as steroids and PUVA.

*Objective*: To describe a variant of AA, Pseudo Alopecia Areata, caused by a particular cupola pin holder (tic-tac) and to offer a non-pharmacological treatment option (NPT).

*Methods*: A prospective open label study in 37 Jewish religious patients (34 males, 3 females, mean 35 ± 2 years), previously diagnosed and treated for scalp AA were randomly referred to one of the three NPT intervention methods: small cupola held by two pins, large cupola held by one pin and similar cupola held by a different pin.

*Results*: Three of the ten patients (33.3%) from the first group developed secondary AA from the additional pin. No changes were seen in the second group. Ten of the seventeen patients (58.8%) from the third group achieved immediate improvement subsequent to replacing the original pin with a new one on a larger cupola.

*Conclusions*: Conservative pharmacological treatment failed to repair the lesions. The addition of a second pin caused an additional lesion. In contrast, replacing the cupola with a larger one and the original pin-fastener with a different type, successfully reduced the lesions.

*Keywords*: Alopecia Areata; Treatment; Antimicrosomal antibody; Autoimmune disease

### INTRODUCTION

Alopecia Areata (AA) is a disease of unknown etiology characterized by hair loss. It can affect men, women or children (Madani and Shapiro, 2000). Although other causes have been suggested, it is widely believed that AA is an autoimmune disease (Sahn, 1995; McElwee *et al.*, 1999; Madani and Shapiro, 2000; Yano *et al.*, 2002) because of the likely presence of antimicrosomal antibody (Ab), antithyroglobulin Ab, antigastric parietal cell Ab and adrenal cell Ab (Paus *et al.*, 1993; McDonagh and Messenger, 1994; Schwartz and Janniger, 1997). There is no conclusive diagnostic test for AA. Dermatologists occasionally rely on clinical findings for diagnosis. Typically, the initial lesion appears as a smooth bald patch. Definitive diagnosis can be made by skin biopsy showing a reduction in hair follicle anagen/telogen phase. Treatment of AA consists of a range of therapies, for which partial success has been claimed. Most of the current modalities used are more effective in the milder forms than in those with extensive hair loss. The various therapies can be divided into several groups:

1. Irritants, such as Dithranol and Phenol (Sherertz and Sloan, 1988; Price, 1999);
2. Immune inhibitors, such as steroids and PUVA (Healy and Rogers, 1993; Whitmont and Cooper, 2003);
3. Cyclosporin A (Moreno *et al.*, 2002);
4. Immune enhancers (Shapiro, 1993; van der Steen and Happle, 1993) such as Inosipley and Thyomopentin and
5. Minoxidil and others (Olsen *et al.*, 1992; Epstein, 1993; Micali *et al.*, 1996; Sharquie and Al-Obaidi, 2002).

Treatment of the concomitant psychopathological disorder has also been suggested (Garica-Hernandez *et al.*, 1999). Positive prognosis is based on the assertion that AA sufferers are capable of re-growing hair even after many years of hair loss. Spontaneous re-growth occurs in 10–30% of cases. In this article, we describe 37 patients

**Financial Disclosure*: This study did not receive any funding from sources other than the Barzilai Medical Center.
[†]Corresponding author. Tel.: +972-8-6745871/2. Fax: +972-8-6745526. E-mail: yosefy@barzi.health.gov.il

ISSN 1740-2522 print/ISSN 1740-2530 online © 2003 Taylor & Francis Ltd
DOI: 10.1080/10446670310001642131

previously diagnosed and treated for AA. The common port of entry for all our patients was the use of a metal clip to hold a skull-cap in place. Skull-caps of various sizes and shapes are widely used in many religions. We describe a new method of treatment for this unique form of AA that we refer to as Pseudo Alopecia Areata (PAA).

### PATIENTS AND METHODS

Thirtyseven healthy white patients (34 males, 3 females) aged 22–52 years (mean 35 ± 6), formerly diagnosed with scalp AA, were selected. Subjects were selected according to their order of appearance. All patients resided in the cities of Ashkelon and Rehovot, in Israel, between the years 1997 and 2000. All were patients of the dermatology departments of the two medical centers. The only reason for them arriving at the dermatology clinic was scalp AA.

The study had been approved by the Institutional Review Board Committee and all subjects gave written informed consent.

Complete clinical and laboratory examinations to eliminate other possible causes for hair loss were conducted. The diagnosis was confirmed histologically, according to established criteria of reduction in the alogen/telogen phase of hair follicle. Damaged hair follicles and inflammatory changes were also seen.

All patients wore a skull-cap which was reinforced to the hair by a metal clip and all had local alopecia characterized by a port of entry part of the bald lesion (Fig. 1). We assumed this remark was caused through recurrent daily trauma by the metal clip (Fig. 2). This port of entry was used as a remark in order to distinguish between AA and PAA. The treatment modalities used were: in 32 patients (86.5%) intradermal steroids and in 5 patients (13.5%) oral steroids. All patients received topical steroids. After 1 month, no improvement was noticed as a result of the above treatments. Thus, three non-pharmacological treatments were randomly introduced (1:1:2 order) in order to reduce the cupola movements and thus the pin trauma, as follows:

1. Two pins, small cupola (10 patients),
2. One pin, large cupola (10 patients) and
3. Different pin, same cupola (17 patients). The different pin was not made of metal and had no sharp edges.

The results were tested for statistical significance using the Student's *t*-test.

### RESULTS

Thirty-seven patients (34 males, 3 females) aged 22–52 years (Mean 35 ± 6) with scalp lesions formerly diagnosed as AA were selected. All failed to recover with topical and intradermal or oral steroid treatments. Non-pharmacological approaches used were the replacement of the original cupola and pin fastener with the following options:

1. Two pins, small cupola (10 patients),
2. One pin, larger cupola (10 patients) and
3. Different pin, same cupola (17 patients).

The first method of fastening a small cupola with two pins, thereby attempting to reduce the cupola movements and thus the pin trauma, failed, and caused a second PAA at the location of the additional pin (Fig. 2) in three of the ten patients (30%). No changes were observed in the second group, where a larger cupola was held in place by one pin. Ten of the seventeen patients from the third group (58.8%), where the original cupola was held in place by a different pin, showed immediate hair re-growth ($P < 0.001$).

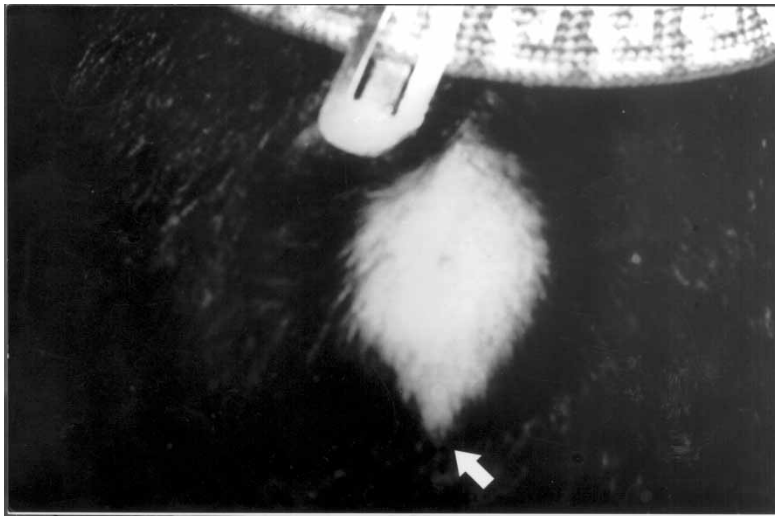

FIGURE 1 Dressing cupola enhanced by pin, developed AA variant (PAA) characterized by a posterior lower port of entry (see arrow) where the pin holder (tic-tac) was inserted and attached.

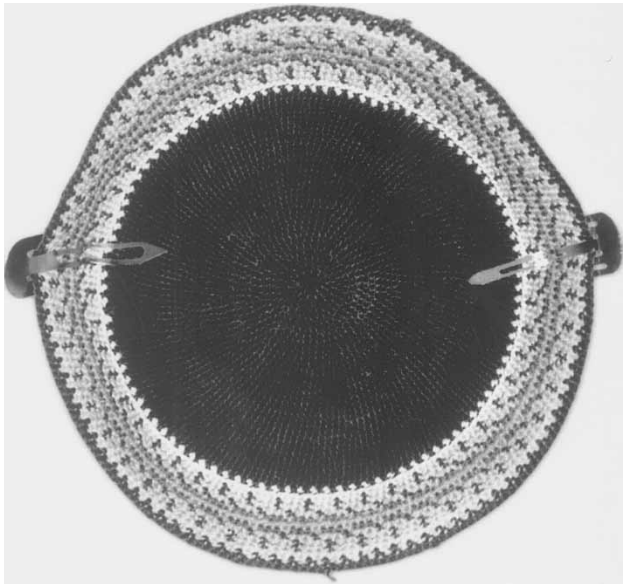

FIGURE 2 The inside part used to hold the cupola with two different types of metal pin holders. Note the sharpness of the metal end.

### DISCUSSION

AA is a disease characterized by hair loss that is often emotionally devastating for patients. Although the cause of AA is unknown, it is widely believed to be an autoimmune disease (McDonagh and Messenger, 1994; Sahn, 1995; Schwartz and Janniger, 1997; McElwee *et al.*, 1999; Madani and Shapiro, 2000; Yano *et al.*, 2002). Treatment of AA consists of various therapies, through which limited success has been achieved. The pharmacological treatment consists of the use of irritants, immune inhibitors, Cyclosporin A, immune enhancers and others (Stroud, 1987; Sherertz and Sloan, 1988; Healy and Rogers, 1993; Paus *et al.*, 1993; McDonagh and Messenger, 1994; Price, 1999; Moreno *et al.*, 2002; Tsai *et al.*, 2002; Whitmont and Cooper, 2003). Spontaneous hair re-growth occurs in 10–30% of cases.

We selected 37 religious patients with a variant of AA that we describe as PAA. All lesions were characterized by a small remark in the posterior-lower part of the lesion. The remark was a result of the recurrent trauma caused by placing and releasing the special pin (tic-tac), and by the continuous trauma caused by the pin's intermittent movements throughout the day.

When the pharmacological treatments failed, we introduced non-pharmacological treatments. The first group used the two pins/small cupola method. This method failed in 67% of patients, and furthermore, 33.3% of patients developed a second PAA scalp lesion. This treatment's failure reinforced our theory that the induction of PAA in these patients was a result of the pin fastener's irritating qualities. When we changed the pin and used a bigger cupola, facilitated hair re-growth was seen. We conclude that in those patients with cupola dressing and with AA, when the lesions are characterized by a port of entry in the posterior-lower part, where the special pin (tic-tac) is inserted, a non-pharmcological approach is more effective.

Although religious women use the same pinholder (tic-tac) to hold their head scarf in place, they are less likely to develop PAA and a male predominance was noted. The reason for this might be the lesser irritation caused by the pinholder in women due to the different location of the pinholders on the scarf (behind the ears) and the greater amount of hair.

From the three methods used, a larger cupola, changing the pin type and careful insertion and removal of the pin, are all recommended.

### *References*

Epstein, E. (1993) "Alopecia areata, topical minoxidil, and balanced reviews [letter: comment]", *Arch. Dermatol.* **129**, 908–909.

Garica-Hernandez, M.J., Ruiz-Dublado, S., Rodriguez-Pichardo, A. and Camacho, F. (1999) "Alopecia areata, stress and psychiatric disorder: a review", *J. Dermatol.* **26**, 625–632.

Healy, E. and Rogers, S. (1993) "PUVA treatment for alopecia areata—does it work? A retrospective review of 102 cases", *Br. J. Dermatol.* **129**, 42–44.

Madani, S. and Shapiro, J. (2000) "Alopecia areata update", *J. Am. Acad. Dermatol.* **42**, 549–566, quiz 567–570.

McDonagh, A.J. and Messenger, A.G. (1994) "The aetiology and pathogenesis of alopecia areata", *J. Dermatol. Sci.* **7**, S125–S135.

McElwee, K.J., Spiers, E.M. and Oliver, R.F. (1999) "Partial restoration of hair growth in the DEBR model for alopecia areata after *in vivo* depletion of CD4+ T cells", *Br. J. Dermatol.* **140**, 432–437.

Micali, G., Cicero, R.L., Nasca, M.R. and Sappupo, A. (1996) "Treatment of alopecia areata with squaric acid dibutylester", *Int. J. Dermatol.* **35**, 52–56.

Moreno, J.C., Ocana, M.S. and Velez, A. (2002) "Cyclosporin A and alopecia areata", *J. Eur. Acad. Dermatol. Venereol.* **16**, 417–418.

Olsen, E.A., Carson, S.C. and Turney, E.A. (1992) "Systemic steroids with or without 2% topical minoxidil in the treatment of alopecia areata", *Arch. Dermatol.* **128**, 1467–1473.

Paus, R., Slominski, A. and Czarnetzki, B.M. (1993) "Is alopecia areata an autoimmune-response against melanogenesis-related proteins, exposed by abnormal MHC class I expression in the anagen hair bulb?", *Yale J. Biol. Med.* **66**, 541–554.

Price, V.H. (1999) "Treatment of hair loss", *N. Engl. J. Med.* **23**(341), 964–973.

Sahn, E.E. (1995) "Alopecia areata in childhood", *Semin. Dermatol.* **14**, 9–14.

Schwartz, R.A. and Janniger, C.K. (1997) "Alopecia areata", *Cutis* **59**, 238–241.

Shapiro, J. (1993) "Topical immunotherapy in the treatment of chronic severe alopecia areata", *Dermatol. Clin.* **11**, 611–617.

Sharquie, K.E. and Al-Obaidi, H.K. (2002) "Onion juice (*Allium cepa* L.), a new topical treatment for alopecia areata", *J. Dermatol.* **29**, 343–346.

Sherertz, E.F. and Sloan, K.B. (1988) "Percutaneous penetration of squaric acid and its esters in hairless mouse and human skin *in vitro*", *Arch. Dermatol. Res.* **280**, 57–60.

Van der Steen, P.H. and Happle, R. (1993) "Topical immunotherapy of alopecia areata", *Dermatol. Clin.* **11**, 619–622.

Stroud, J.D. (1987) "Diagnosis and management of the hair loss patient", *Cutis* **40**, 272–276.

Tsai, Y.M., Chen, W., Hsu, M.L. and Lin, T.K. (2002) "High-dose steroid pulse therapy for the treatment of severe alopecia areata", *J. Formos. Med. Assoc.* **101**, 223–226.

Whitmont, K.J. and Cooper, A.J. (2003) "PUVA treatment of alopecia areata totalis and universalis: a retrospective study", *Australas. J. Dermatol.* **44**, 106–109.

Yano, S., Nakamura, K., Okochi, H. and Tamaki, K. (2002) "Analysis of the expression of cutaneous lymphocyte-associate antigen on the peripheral blood and cutaneous lymphocytes of alopecia areata patients", *Acta Dermato-Venereol.* **82**, 82–85.

